# A long way from home: Biosecurity lessons learnt from the impact of La Niña on the transportation and establishment of tropical portunid species

**DOI:** 10.1371/journal.pone.0202766

**Published:** 2018-08-22

**Authors:** Matthew J. Hewitt, Mathew Hourston, Justin I. McDonald

**Affiliations:** 1 Department of Primary Industries and Regional Development, Aquatic Biosecurity Surveillance, Perth, Western Australia, Australia; 2 Department of Primary Industries and Regional Development, Bycatch and Harvest Strategies, Science and Resource Assessments Division, Perth, Western Australia, Australia; Duke University Marine Laboratory, UNITED STATES

## Abstract

Marine ecosystems can be modified and shaped by irregular interannual variations in oceanic current patterns and temperatures, such as El Niño and La Niña. These large scale oceanic events have also been shown to influence environmental stressors such as invasive marine species (IMS). Our study indicates that there is a causative link between these climatic events, and atypical detections of native and IMS. Significant La Niña events between 1970 and 2014 were associated with sightings of tropical crab species in temperate waters following a lag period of 18–24 months from the onset of the event. We identified a total of 72 records of six tropical portunid crabs species (from both *Charybdis* and *Scylla*) in temperate waters of south-western Australia following these La Niña events, based on reports in published scientific literature, grey literature and museum records, as well as citizen science networks such as FishWatch and PestWatch apps. We suggest that La Niña conditions facilitated transportation and temporary establishment of crab larvae from their native tropical habitat to temperate regions. As the strength of La Niña events is likely to increase into the future due to the escalating effects of climate change, it is likely that there will be a growth in associated atypical establishment events of IMS. Consequently, biosecurity managers will need to reprioritise resources in order to accommodate the potential impacts of these large scale oceanic events as part of their surveillance programmes.

## Introduction

Irregular interannual variations in oceanic current patterns and temperatures are such influential events that they can shape ecosystems worldwide. These climatic events are responsible for global-scale changes in rainfall distribution, severe weather events, oceanic upwelling intensities, levels of primary production and distribution of marine fauna [1,2]. Climatic events of this magnitude can also influence environmental stressors such as invasive marine species (IMS).

IMS are a major global threat to marine ecosystems and are regarded as a driver of biodiversity loss. They have the potential to significantly and permanently alter natural ecosystems, with economic costs associated with the damage caused by IMS estimated in the tens of millions of dollars [3,4]. The cumulative effects of irregular variations in ocean temperature and current changes aid in the expansion and establishment of marine species into areas where they have previously been absent [5,6]. When the effects of irregular interannual variations in oceanic current patterns and temperatures are coupled with the impact of establishment of IMS, the resulting impact on prevailing marine environmental conditions may be dramatic.

The El Niño Southern Oscillation (ENSO) is an irregular, episodic change in sea surface temperatures (SSTs) and wind patterns, which can significantly influence the Southern Hemisphere. The varying strengths of ENSO phases are given by the Southern Oscillation Index (SOI), which is calculated from the atmospheric pressure differences between Tahiti (French Polynesia), and Darwin (Australia) [7]. Sustained negative SOI values are known as an El Niño phase, which is characterised by sustained warming of the central and eastern tropical Pacific Ocean. Conversely, sustained positive SOI values indicate a La Niña phase [7]. Characterised by a cooler Pacific Ocean, in Western Australia the La Niña phase results in a relatively stronger Leeuwin Current. The Leeuwin Current is a tropical southward-flowing boundary current, extending for 5,500 km off the coast of Western Australia. The strength of this current fluctuates with season (strongest in April–September), but it is also intensified by La Niña conditions, during which a warmer and faster current than usual flows southwards, resulting in higher SSTs. In an El Niño year, the Leeuwin Current becomes weaker and SSTs fall [8]. With the growing influence of climate change, El Niño and La Niña events are predicted to become more frequent and extreme [1,9], which are predicted to lead to changes in ecosystem distribution, structure and functions, such as an increase in mass coral mortalities [10] and aid in range shifts of key habitat-modifying organisms [11].

In November 2010 –April 2011 a “marine heatwave” occurred off the coast of Western Australia during which sea surface temperatures peaked at 3°C above monthly mean. This was caused in part by a very intense La Niña event [12]. The 2010–2011 La Niña event was followed in 2011–2012 with a second positive SOI year, which officially extended the duration of the heatwave event to 24 months. As well as an increase in temperature, the La Niña event also resulted in one of the strongest Leeuwin Currents on record (August 2010-May 2012) [13]. As a direct product of the 2010–2011 heatwave and fast flowing Leeuwin Current, many atypical ecological events were recorded, including migration or recruitment of tropical marine species to latitudes well south of their typical distributions [14,15].

One to two years following the marine heatwave and strong Leeuwin Current event, *Charybdis japonica* (A. Milne-Edwards, 1861), an IMS originating in South East Asia, was detected in both the Peel-Harvey (32°36'59.99" S, 115°38'59.99" E) and Swan River estuaries (32°1'44.66"S, 115°46'57.65"E), Western Australia. As *C*. *japonica* is considered an invasive marine pest in Australia, intensive delimiting activities were conducted to determine the extent of the incursion, a full account of which is presented in Hourston *et al*. [16]. The delimiting and public awareness activities also produced reports of several tropical portunid crab species (family Portunidae) not typically recorded in temperate areas of South-Western Australia (SWA). For several of these species, these were their first recorded sighting in temperate SWA, prompting further investigation of historical atypical sightings of similar species.

In this study we investigated patterns of contemporary and historical reports of tropical portunid crabs in temperate Western Australia and their relationship to El Niño Southern Oscillation cycles. Exploration of the interaction between irregular variations in ocean temperature and current changes and the establishment of IMS may prove important to IMS management into the future, particularly in light of the growing influence of climate change on marine systems.

## Methods

### Collection of historical and contemporary crab species reports

Both historical and contemporary atypical reports of crab species in SWA were included in this investigation. Atypical records were considered to be detections of species outside their documented historical range. The databases Web of Knowledge, Google Web and Google Scholar were searched for records of crab species in SWA using a variety of the terms including; “Western”, “Australia”, “crab”, “distribution”, “portunidae”, “Charybdis”, “range”, “extension”, “ENSO”, “IMS”, “tropicalisation” and “biosecurity”. All reports of crab species (Infraorder, *Brachyura*) were included, while other decapods, such as shrimp and hermit crabs, were not considered in the analysis. Historical reports were drawn from published scientific literature, grey literature and museum records. Reports of atypical species were collated and verified through networks such as the Department of Primary Industries and Regional Development’s (DPIRD) FishWatch and PestWatch apps, set up for reporting marine pests or unusual species of interest [16]. Contemporary reports included in the data set were limited to those that could be verified through the Western Australian Museum, and DPIRD FishWatch and PestWatch apps.

Monthly SOI values sourced from the Australian Bureau of Meteorology were used to collate ENSO information. Official La Niña and El Nino periods and strengths were provided by the Australian Bureau of Meteorology, and were determined by the Bureau’s climatologists using a series of decision rules [7]. The data series was limited to 1970 to correspond with time period of the earliest historical atypical report of a portunid species.

## Results

A total of 72 records of atypical portunid crabs, spanning six species, was identified from both historical and contemporary sources. Contemporary records showed that thirty four individuals were detected during the more recent 2012–2014 biosecurity delimiting activities [16]. Four were identified as the IMS *Charybdis japonica*, while the remaining 30 were native Australian species. Individuals of the native species included four *Charybdis annulata* (Fabricius 1798), four *Charybdis granulata* (de Hann 1833), 14 *Charybdis feriata* (Linnaeus 1758), five *Charybdis natator* (Herbst 1794), and three *Scylla serrata* (Forskål 1775).

Historical records revealed that thirty two individuals of *S*. *serrata* were reported by Gopurenko *et al*. [17] from 2001–2002, documenting a recent colonisation event in south Western Australian estuaries. Isolated museum records and grey literature publications documented a further seven reports between 1971 and 1991. One *C*. *natator* in 1971[18], three *C*. *feriata* in 1972 (A Hosie pers. comm.), one *C*. *feriata* in 1990 and two *C*. *granulata* in 1990 and 1991 (17, A Hosie pers. comm.), which were found in various locations in SWA (Table 1). Reports for *C*. *japonica* and *C*. *annulata* represent the first records for these species in SWA, while for *C*. *feriata*, *C*. *natator* and *C*. *granulata* these are the first records of more than one individual in that same region. Although the detection of the IMS *C*. *japonica* stimulated the investigation of this study, its transportation vector is outside the scope of this paper.

**Table 1:** Species synthesis table of atypical tropical portunid crab species recorded in South-Western Australia from the 2012–2014 *Charybdis japonica* surveillance campaign and from historical records. Known distributions and basic biological data are also presented.

		*Charybdis feriata*	*Charybdis natator*	*Charybdis granulata*	*Charybdis annulata*	*Scylla serrata*
Existing Range						
	Global	Indo-Pacific, Japan, China, Taiwan, Hong Kong,	Indo-Pacific, Egypt, China, Hong Kong	Indo-Pacific, Japan, Taiwan, Hong Kong	Indo-Pacific, South Africa, India, Japan, French Polynesia	Indo-Pacific, Japan, Fiji, Samoa, USA^a^
	Within Australia	NT, QLD, NSW, VIC, WA (North of Shark Bay), South West isolated records	QLD, NSW, WA (North of Shark Bay)	QLD, NSW, WA (North of Shark Bay)	QLD	NT, QLD, NSW, WA (North of Shark Bay, South West isolated records)
Atypical Reports						
	Locality of report	South West (Swan River to Peel-Harvey Estuary)	South West (Swan River to Cockburn Sound)	South West (Hillarys to Peel-Harvey Estuary)	Central WA (Kalbarri, Geraldton)	South West (Fremantle to Albany)
	Contemporary (2012–2014)	14	5	4	4	3
	Historical (1970–2012)	5 (1973,1990)	1 (1971)	1(1991)	0	32 (2001–2002)
Biological Data						
	Max carapace width	200mm	170mm	100mm	85mm	280mm
	Temperature range	13.1°C– 33°C *	13.1°C– 31.1°C *	13.1°C– 31.1°C *	13.1°C– 31.1°C *	12.0°C– 35.0°C
	Known salinity range	Marine, Estuarine	Marine, Estuarine	Marine, Estuarine	Marine, Estuarine	2.0–40.0 ppt
	Habitat	Mud, sand, rock, coral reef flats and estuaries	Mud, sand, rock, coral reef flats and estuaries	Sand, rock, and estuaries	Mud, sand, rock, tide pools, reef flats and estuaries	Intertidal mangroves, mud, sand, river banks and estuaries
	Depth	Sublittoral, 60m	Sublittoral, 60m	Sublittoral, 35m	Littoral and sublittoral, 20m	Littoral and sublittoral, 15m
	Detected IMS	No	No	No	No	Yes (Hawaii, Florida)
Key References		[19–26]	[20–25,27,28]	[19,20,21,23,25,29]	[20,24,25,30]	[17,20–22,24,31]

* Temperature ranges are based on observed water temperatures (obtained from NOAA) from regions where populations are present in the absence of studies obtained from the literature.

^a^ Existing range of *S*. *serratta* in the USA are known introductions in Hawaii and Florida.

**Fig 1:**
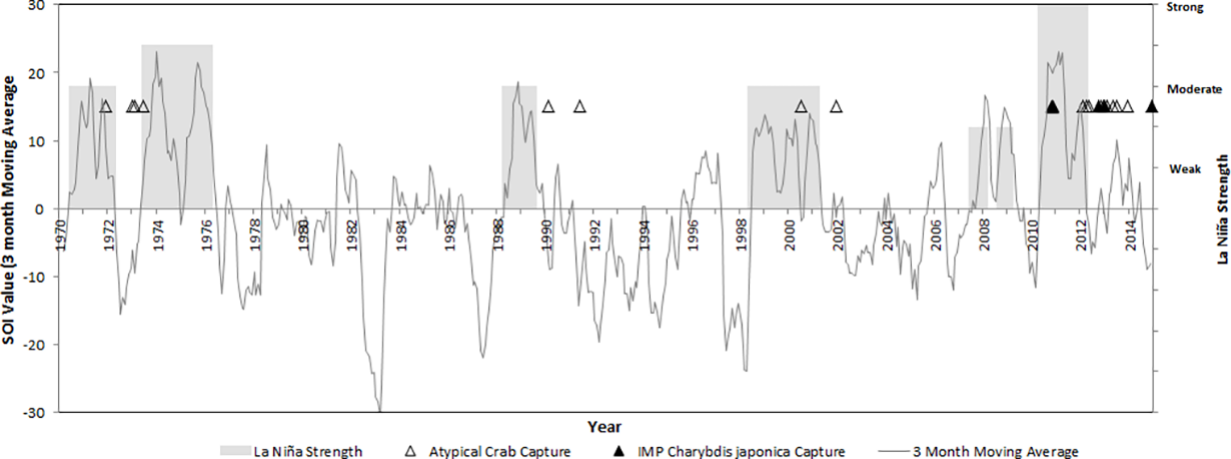
Southern Oscillation Index (SOI) time series between 1970 and 2014. La Niña events (grey highlight) and their strength, as indicated by the Bureau of Meteorology, since 1970. Captures of tropical portunids in South-Western Australia indicated by hollow triangles and sightings of *Charybdis japonica* are indicated by black triangles.

Monthly SOI values, and official La Niña periods and strengths fluctuated between 1970 and 2014 (Fig 1). There was a clear pattern between atypical climatic events and atypical crab detections in SWA. Comparing the timing of the 72 reports of tropical portunid crabs in SWA and the time series of the Southern Oscillation Index between 1970 and 2014, the reports of atypical tropical crabs begin approximately 18–24 months after the onset of significant La Niña conditions. Four of the five ‘Moderate to Strong’ La Niña periods since 1970 were followed by a report an atypical crab species, following a lag of approximately 18–24 months (Fig 1). The initial detection of *C*. *japonica* in November 2010 may have possibly followed the ‘Weak to Moderate’ 2008–09 La Niña event however the transportation vector of this IMP is outside the scope of this paper. Only the ‘Weak to Moderate’ La Niña 2007 event and the ‘Strong’ La Niña 1973–1976 event were not followed by atypical crab reports (Fig 1).

## Discussion

Our study indicates that irregular interannual variations in oceanic current patterns and temperatures may impact upon the establishment of IMS. Onset of significant La Niña events between 1970 and 2014 prompted atypical sightings of tropical crab species in the temperate waters of SWA 18–24 months after the onset of the events. As the effects of La Niña in Western Australia comprise increased sea surface temperatures and increased southerly flow of the Leeuwin Current along the coast, it is likely that the La Niña conditions both facilitated the transportation as well as the establishment of tropical crab larvae from tropical to temperate regions.

The 18–24 month delay between the onset of La Niña and the first reports of crabs likely represents the time between larval settlement and the size at which the adult crabs were large enough to be caught by fishers. Gopurenko *et al*.[17] reached a similar conclusion when considering the 32 reports of *Scylla serrata* in SWA estuaries between 2001 and 2002. Gopurenko *et al*.(15) proposed that a stronger Leeuwin Current flow had successfully transported larvae to southern waters and temporarily provided favourable conditions for larval settlement and growth. In 2001 conditions returned to a neutral SOI, a weaker Leeuwin Current and cooler SSTs, at which permanent populations did not establish in SWA [32]. While the mechanisms behinds the failure to establish were not investigated it was predicted that larval supply decreased or ceased and the individuals that had initially settled were able to live and grow but not able to create a self-sustaining population.

While changing environmental conditions may provide ideal conditions for adaptable and resilient species, for many species the marine heatwave was detrimental, and caused significant ecosystem-level stress [15,33]. As stressed and degraded ecosystems are often susceptible to IMS colonisation [34,35], conditions such as these provide ideal opportunities for new IMS to gain a foothold in their new environment and proliferate [36]. This is the situation that occurred in 2010–2012 in which three tropical IMS were detected in temperate SWA; *C*. *japonica* [16], *Didemnum perlucidum* [37] and *Perna viridis* (spawned inside a vessel only) [38]. Examples also exist in the northern hemisphere where the expansion of transport of *Pleuroncodes planipes*, [39] and the range expansion of the known invasive species *Carcinus meanas* and *Rhithropanopeus harrisii*, in the North East Pacific [6,40] were linked to a combination of strong ENSO indices, elevated water temperatures and weakened southward shelf currents.

With the escalating effects of climate change, El Niño and La Niña events are predicted to become more extreme [1,9], intensifying stress on marine systems, and in turn increasing the risk of IMS establishment success. Many poleward marine species range shifts have already been documented, with distribution models indicating further expansion and tropicalisation across a wider range of species [41–44]. The implications for marine biosecurity and the spread of IMS are clear. IMS mitigation activities must be predictive, targeted and efficient. The use of environmental indicators, such as SSTs, SOIs and the strength of oceanic currents, may aid in prioritising key biosecurity management strategies and enabling well informed decisions regarding IMS.

The link between irregular interannual variations in oceanic current patterns and temperatures and the establishment of IMS suggests that biosecurity researchers and managers can potentially exploit the forecasting ability of major climatic indices, such as the SOI, to better inform the allocation of biosecurity efforts using a risk-based framework. For example, a species-based approach, such as targeting (sub)tropical organisms in temperate environments during or after strong La Niña events may prove a useful management strategy. Efficiencies may be gained by targeted monitoring of priority species, locations and periods based on SOI, SSTs or other indicators of environmental change or stress. Considering the 18–24 month delay in crab detections observed in this study, opportunity may exist for early detections methods, such as propagule sampling, or allow a lead-in period in which to efficiently redeploy monitoring efforts and Citizen Science programs targeted at an appropriate species suite.

Targeting key habitats and regions for IMS monitoring can also be prioritised during times of susceptibility. Areas of low biodiversity, threatened habitats or those subjected to a high level of disturbance would be the most vulnerable during times of environmental anomalies, such as those brought on by particularly strong La Niña periods. Estuaries or semi-enclosed coastal water bodies in particular provide ideal environments for IMS to establish and persist as they are often the centre of anthropogenic disturbance through pollution, habitat loss, altered hydrology or overfishing and the focal point of multiple aquatic transportation vectors [45–48]. Estuaries in SWA are particularly vulnerable to elevated changes in temperature and climate change, known to be an ocean warming hotspot [44]. Elevated temperatures can have both positive and negative effects on species in SWA estuaries, however it is known to negatively affect species physiology and performance that are already close to their thermal maximum [44]. Such changes in community composition of estuarine species provide a window where IMS monitoring should be concentrated.

Our study suggests there is a link between irregular interannual variations in oceanic current patterns and temperatures, and the detection of IMS. Significant La Niña events between 1970 and 2014 were associated with sightings of atypical tropical crab species in the temperate waters of SWA following a lag period of 18–24 months. While reports of a species do confirm its presence, the converse is not necessarily true i.e. a lack of reports does not prove their absence. It is likely that La Niña conditions facilitated an increase in transportation and establishment of tropical crab larvae from tropical to temperate regions. As the strength of La Niña events are likely to increase into the future due to the growing effects of climate change, it is possible that there will be a rise in associated atypical establishment events of tropical native and IMS. At present, the elevated SSTs experienced during the height of the La Niña phases are temporary. Consequently, habitat, nutrient and reproductive conditions are likely to remain unfavourable for ongoing recruitment and permanent establishment of tropical IMS in temperate regions. This may change however, as oceanic current patterns and temperatures continue to change at a global scale, the likelihood of permanent range shifts and proliferation of IMS continues to increase. As such, biosecurity mangers will need greater predictive modelling to anticipate IMS range shifts and to improve early detection and subsequent management of tropical IMS entering atypical coastal regions.
